# IBA and melatonin increase trigonelline and caffeine during the induction and initiation of adventitious roots in *Coffea arabica* L. cuttings

**DOI:** 10.1038/s41598-023-41288-x

**Published:** 2023-09-13

**Authors:** Francisco Hélio Alves de Andrade, Ana Maria Oliveira Ferreira, Lillian Magalhães Azevedo, Meline de Oliveira Santos, Gladyston Rodrigues Carvalho, Mário Lucio Vilela de Resende, Elisa Monteze Bicalho, Vânia Aparecida Silva

**Affiliations:** 1 Federal University of Lavras, Lavras, Minas Gerais Brazil; 2 Agricultural Research Company of Minas Gerais, Lavras, Minas Gerais Brazil; 3 Scholarship BDCTI-I, FAPEMIG/INCT Café, Lavras, Brazil; 4 Scholarship PQ, CNPq, Lavras, Brazil; 5 Scholarship DT, CNPq, Lavras, Brazil

**Keywords:** Biochemistry, Biotechnology, Plant sciences

## Abstract

Caffeine and trigonelline are found in *Coffea arabica*, and show antioxidant roles and growth and development functions. However, there are no reports on trigonelline and caffeine in relation to coffee rooting. The aim was to evaluate the impact of application of indole-3-butyric acid (IBA) and melatonin on caffeine and trigonelline at different stages of adventitious rooting in cuttings. In addition, to study the correlation between these metabolites and H_2_O_2_, phenols, and antioxidant enzymes. Four treatments (Control, melatonin 21 µM (M21), melatonin 43 µM (M43), and IBA 7380 µM (IBA)) were used, with four replications. The growth and biochemical parameters of the antioxidant system were performed in induction, initiation, and extension rooting stages. Higher concentrations of trigonelline and caffeine quantified in the induction and initiation stages were positively correlated with higher percentage of rooted cuttings. Trigonelline and caffeine were positively correlated with H_2_O_2_ in all stages of development of adventitious roots. The correlations of trigoneline and caffeine with phenols and antioxidant enzymes reveal different profiles, depending on the phases. The results indicate that IBA and melatonin increase trigonelline and caffeine during the induction and initiation of adventitious roots in *Coffea arabica* cuttings, which is correlated with a higher percentage of rooted cuttings.

## Introduction

Coffee is the most consumed beverage worldwide. Brazil is one of the largest producers, and *Coffea arabica* L. constitutes an important species in the Brazilian agronomic and economic scenario. To assure quality combined with high yields in the field, it is necessary to carefully choose the coffee seedlings and plantlets produced. Vegetative propagation makes it possible to establish coffee plantations with high yield of beans that provide a quality beverage through selection of superior clones. Therefore, the production of seedlings is the first important step to assure coffee production. In the selection phase of the *Coffea* breeding program with the aim of making a clonal cultivar available, asexual propagation is important to ensures that all the new plants produced are genetically identical to the selected parent plant. Clonal propagation helps preserve and stabilize these intricate genetic compositions, ensuring that the unique gene combinations of the selected genotypes are retained without recombination through sexual reproduction. Coffee plants grown from seeds take a relatively long time to reach maturity and produce coffee cherries. In contrast, vegetative propagation allows breeders to rapidly multiply the selected genotypes, significantly reducing the time required to produce large quantities of clonal coffee plants. This accelerates the development and release of improved cultivars, with more rapid benefits for farmers and consumers.

However, the rooting capacity of the *C. arabica* plant is an obstacle to plant production by the traditional methods of propagation through cuttings. Therefore, a consistent and accurate vegetative method for coffee plantlets production has not yet been achieved. There is wide variation regarding the rooting capacity among *C. arabica* cultivars, in addition to genetic variation in the concentration of secondary metabolites^1^, such as phenolics, and nitrogen compounds. The effect of secondary metabolites as antioxidant molecules and in protection of auxins against decarboxylation has already been confirmed^2^; therefore, they may be associated with adventitious rooting. Alkaloids (nitrogen compounds), such as trigonelline and caffeine, are important components in of *C. arabica* beverages quality^3^. Yet, there is no information about the role of caffeine and trigonelline in the development of adventitious roots in *C. arabica*.

Caffeine (1,3,7 N-methylxanthine) and trigonelline (1 N-methylnicotinic acid) are secondary metabolites - alkaloids - derived from purine and pyridine nucleotides, respectively^3^. The concentrations vary depending on the type and age of the plant organ. Trigonelline is found in all organs of *C. arabica*, but in higher concentrations in young stems and in areas with active meristems. Caffeine is found in greater amount in cotyledons and leaves, with low concentration in young stems^3,4^.

Some studies associate these secondary compounds in coffee beans with variations in beverage quality, due to their antioxidant properties^5–7^. Caffeine and trigonelline have also been reported as functioning in antioxidant defense of plants under stress and against pathogens and herbivores^8–13^. In particular, trigonelline can be considered a reserve compound for the biosynthesis of nicotinamide adenosine dinucleotide (NAD) during imbibition and germination of *C. arabica* seeds^14^. The detoxification of nicotinic acid is particularly necessary, as it is toxic to the plants in high concentrations^15^. In mungbean (*Phaseolus aureus*), trigonelline was found in high concentration in the embryonic axis during germination^16^.

Three stages of rooting are considered in *C. arabica* cuttings, namely, induction, initiation, and extension of the root primordia. The induction phase involves biochemical events preceding visible morphological changes, whereas initiation comprises the beginning of primordia and meristems; extension is understood as intra-cauline growth of the root primordia and root emergence^17–19^. Multiplication of the coffee cuttings  also requires the application of growth regulators in most cases. The most used in *Coffea arabica* plant cuttings is indole-3-butyric acid (IBA). More recently, melatonin has also been proposed as an alternative for pomegranate woody cuttings^20,21^. Melatonin is another growth regulator that is involved in root formation, acting like auxin in rooting and root growth in different plant species^22^. In addition, the application of melatonin and auxin in *Leucojum aestivum* cultivated in vitro stimulated alkaloid biosynthesis^23,24^.

Thus, this paper raises the hypothesis that caffeine and trigonelline are involved in the rooting of *Coffea arabica* plants when treated with IBA and melatonin. The aim was to evaluate the impact of application of IBA and melatonin on caffeine and trigonelline at different stages of adventitious roots in cuttings. For that purpose, we evaluated the correlation between the concentrations of trigonelline and caffeine at the base of cuttings treated with IBA and melatonin and evaluated the formation of adventitious roots in cuttings of *Coffea arabica* plants. We also studied the correlation between these metabolites and H_2_O_2_, phenols and antioxidant enzymes.

## Results

### Morphology and rooting as a function of melatonin and exogenous IBA

**Figure 1:**
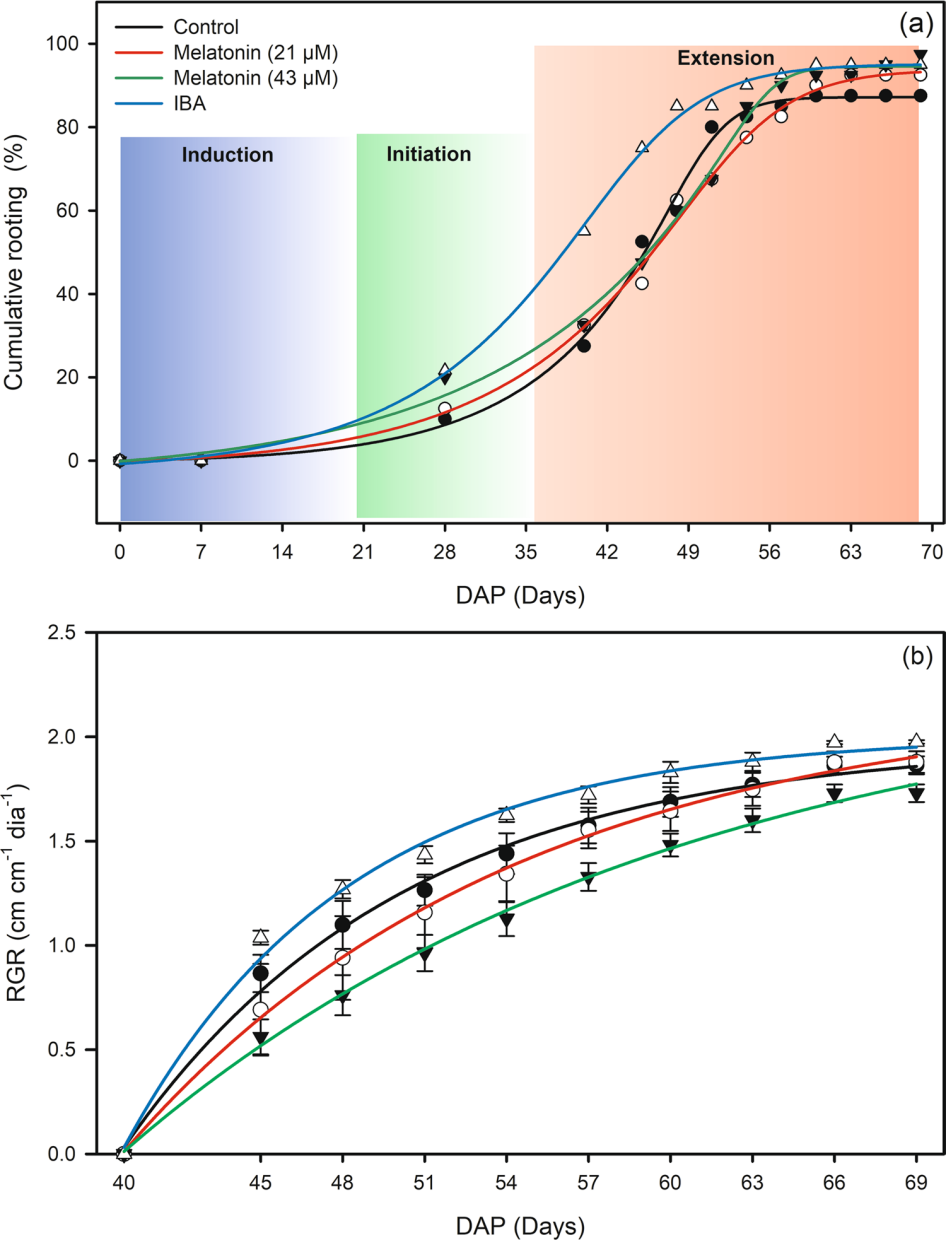
Cumulative rooting and rooting phases (**a**), and *RGR* relative growth rate (**b**) of *Coffea arabica* L. cuttings under application of melatonin and IBA. The induction phase is represented by the blue gradient, the initiation phase by the green gradient and the extension phase by the red gradient.

The cumulative percentage of rooted cuttings (PRC) was fitted to sigmoid function with a R^2^ of 0.99 (Table 1). The result in Fig. 1a shows that exogenous application of IBA accelerated the rooting process. At 47 days after planting (DAP), the cuttings that  received the IBA treatment had a higher PRC at 82%, whereas M21, M43, and the control did not exceed 58%. Stabilization of the IBA, M43, and control treatments, however, occurred at 59 DAP, with values of 94, 94 and 87%, respectively. The relative growth rate (RGR) was fitted to the exponential function with a R^2^ of 0.99 for all treatments. It was clear that IBA led to a higher RGR than that of the other treatments (Fig. 1b). All equations showed a significant effect at p < 0.01.

**Table 1:** Equations referring to Fig. 1(a) and (b).

Treatment	Equation	*p*-value
Percentage of rooted cuttings		
Control	ŷ = − 0.54 + 87.73/(1 + exp(− (x − 50.56)/ 2.09))^0.21^ R^2^ = 0.99	0.0001
M21	ŷ = − 1.81 + 95.59/(1 + exp(− (x − 53.07)/ 4.04))^0.31^ R^2^ = 0.99	0.0001
M43	ŷ = − 4.05 + 98.60/(1 + exp(− (x − 55.95)/ 1.55))^0.09^ R^2^ = 0.99	0.0001
IBA	ŷ = − 2.98 + 98.09/(1 + exp(− (x − 44.52)/ 4.42))^0.37^ R^2^ = 0.99	0.0001
Relative growth rate		
Control	ŷ = − 94.17 + 96.14(1 − exp(− 0.0976x)) R^2^ = 0.99	0.0001
M21	ŷ = − 33.25 + 35.45(1 − exp(− 0.0697x)) R^2^ = 0.99	0.0001
M43	ŷ = − 14.37 + 16.70(1 − exp(− 0.0494x)) R^2^ = 0.99	0.0001
IBA	ŷ = − 265.74 + 267.75(1 − exp(− 0.1227x)) R^2^ = 0.99	0.0001

**Figure 2:**
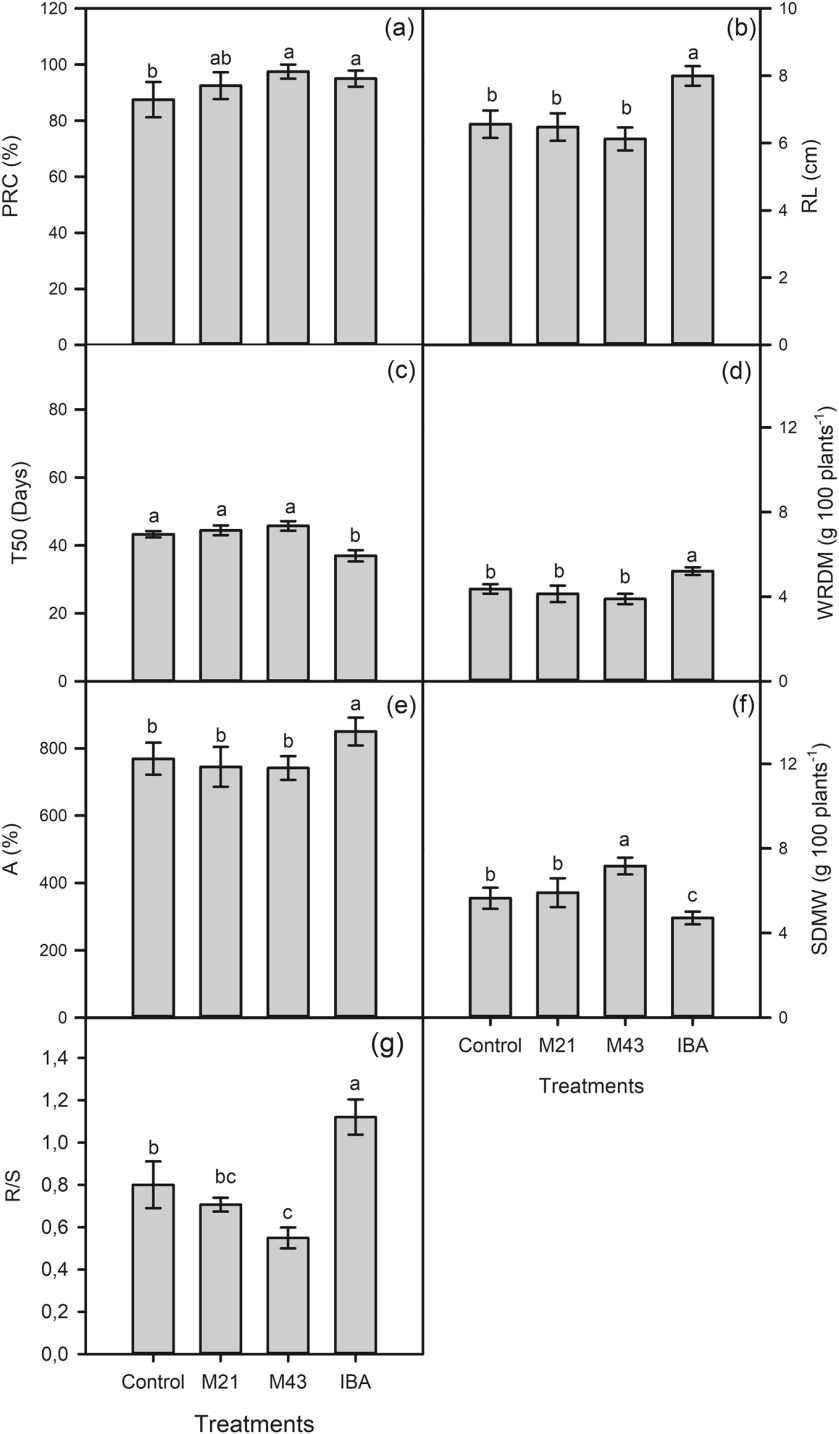
*PRC* percentage of rooted cuttings (**a**), *RL* root length (**b**), *T50* time required to reach 50% of maximum potential (**c**), *WRDM* weight of root dry matter (**d**), *A* cumulative rooting area (**e**), *SDMW* shoot dry matter weight (**f**) and *R*/*S* root/shoot dry matter ratio (**g**) as a function of exogenous application of melatonin and IBA in *Coffea arabica* plant cuttings.

**Figure 3:**
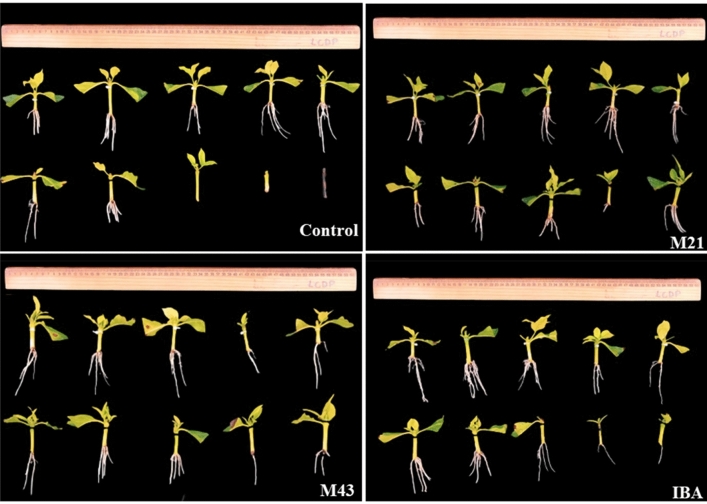
Photographs of control and treated *Coffea arabica* cuttings (M21, M43, and IBA) at 69DAP.

The data showed that the application of melatonin and IBA led to a significant effect by the Duncan test at 5% probability. Melatonin M43 increased the PRC by 11% and IBA by 8.6% in relation to the control (Figs. 2a, 3).

Root length (RL), root dry matter weight (WRDM) and cumulative root area (A), in turn, were positively affected by the application of IBA, which resulted in notable increases of 1.4 cm, 0.84 g, and 81%, respectively, in relation to the control (Fig. 2b,d,e). The IBA at the concentration of 1500 ppm had lower T50 (Fig. 2c). Melatonin M43 is also involved in shoot formation, as SDMW was positively affected by the application of M43, with an increase of 1.52 g in relation to the control (Fig. 2f). The root/shoot dry matter ratio (RS) was higher when IBA was applied, while the lowest value was found in the application of M43 (Fig. 2g), due to the higher SDMW caused by the treatment (Fig. 2f).

### Changes in enzymatic activity, H_2_O_2_ content and phenols during the rooting phases

**Figure 4:**
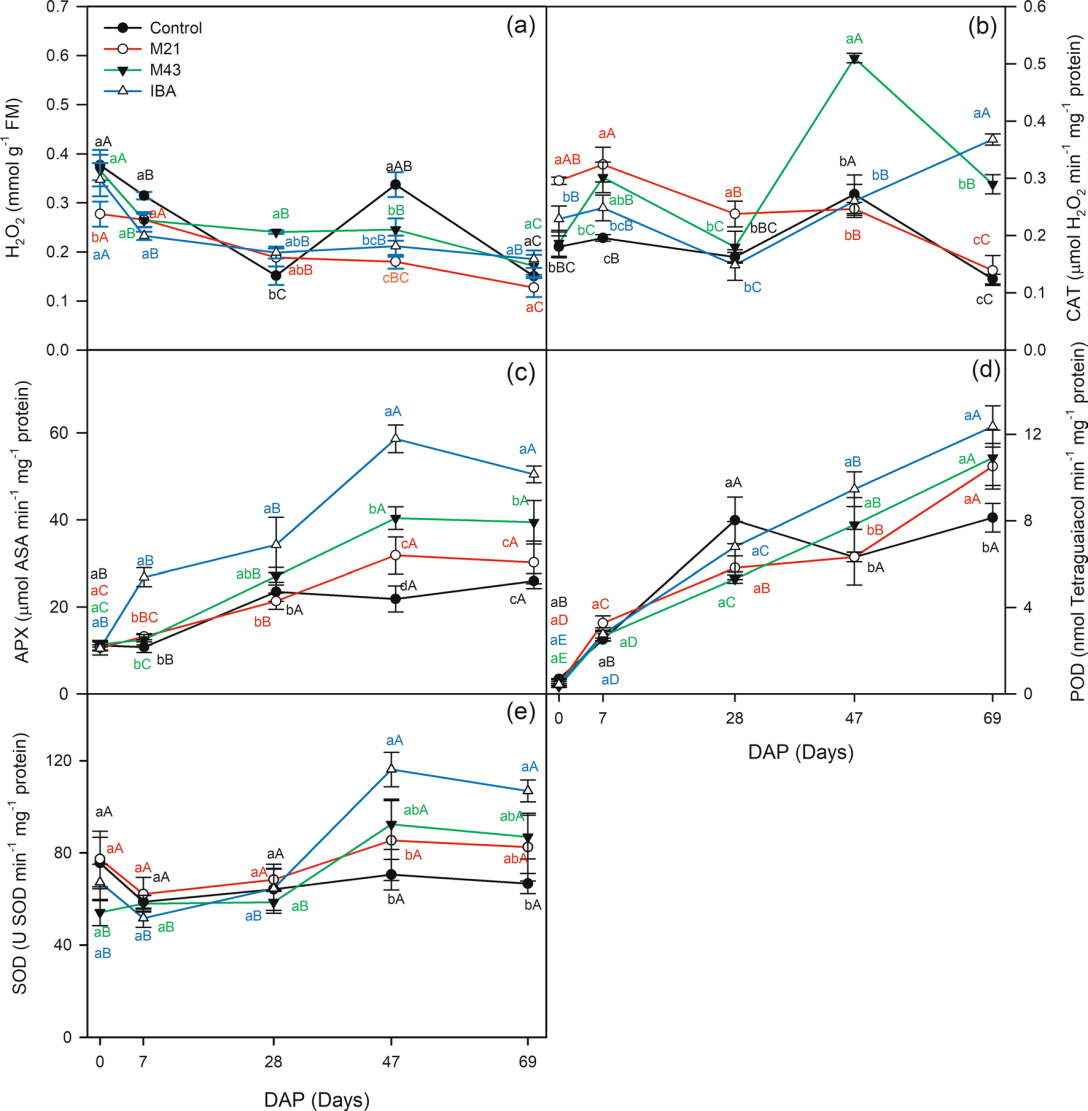
*H*_2_*O*_2_ hydrogen peroxide (**a**), *CAT* catalase (**b**), *APX* ascorbate peroxidase (**c**), *POD* peroxidase (**d**), *SOD* superoxide dismutase (**e**), depending on the application of melatonin and IBA at 0, 7, 28, 47 and 69 DAP (days after planting) in cuttings of *Coffea arabica* cultivar Catuai IAC-144. Lowercase letters compare the stimulants (melatonin and IBA) within each day after planting (DAP). Uppercase letters compare the DAP within each stimulant by Duncan’s test (*p* < 0.05).

**Figure 5:**
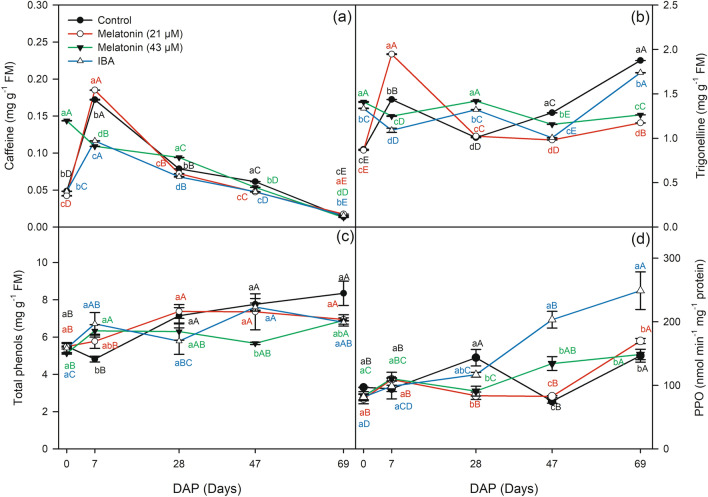
Caffeine (**a**), trigonelline (**b**), total phenols (**c**), and *PPO* polyphenoloxidase (**d**) as a function of melatonin and IBA application at 0, 7, 28, 47 and 69 DAP (days after planting) in *Coffea arabica* cuttings. Lowercase letters compare the stimulants (melatonin and IBA) within each day after planting (DAP). Uppercase letters compare the DAP within each stimulant by Duncan’s test (*p* < 0.05).

During the rooting phases, significant changes were observed in enzymatic activities and content of H_2_O_2_, total phenols, and alkaloids by the Duncan test at 5% probability (Figs. 4, 5). The application of IBA, M21 and M43 led to a reduction in the H_2_O_2_ content at 7 DAP of 26%, 15% and 16%, respectively, compared to the control (Fig. 4a).

In the initiation phase at 28 DAP, the data showed that the application of IBA, M21, and M43 stimulated an increase in the H_2_O_2_ content of 31%, 20% and 58%%, respectively, compared to the control. Catalase (CAT) activity was positively affected by the application of melatonin and IBA at different rooting stages (Fig. 4b). Exogenous application of M21 melatonin resulted in values that were 64%, 65% and 46% higher than the control at 0 DAP in induction and initiation. M43, in turn, caused an increase in CAT activity of 58%, 87% and 133% compared to the control in induction and in extension at 47 and 69 DAP, respectively. IBA let to 27%, 27%, and 197% greater CAT activity than that of the control at 0, 7 and 69 DAP, respectively.

Application of IBA positively affected ascorbate peroxidase (APX) activity, which had values that were 16, 11, 37, and 23 µmol ascorbic acid min^−1^ mg^−1^ higher than the APX activity of the control at 7, 28, 47, and 69 DAP, respectively (Fig. 4c). Application of M43 on the cuttings of the Catuai IAC-144 cultivar also brought about greater APX activity compared to the control in the extension phase at 47 and 69 DAP.

The IBA and M43 treatments significantly affected peroxidase (POD) activity at 47 and 69 DAP (Fig. 4d). POD activity increased 50% with IBA and 23% with M43 at 47 DAP, 52 and 34% at 69 DAP compared to the control. The result of superoxide dismutase (SOD) activity was significantly affected according to the Duncan test at 5% probability (Fig. 4e). The highest SOD activities were reported in the IBA treatment in the root extension phase at 47 and 69 DAP.

Phenolic compounds, alkaloids, and polyphenoloxidase (PPO) activity were affected by the application of melatonin and IBA at different rooting stages (Fig. 5).

Melatonin M43 brought about an increase of 197% in caffeine content at 0 DAP and an increase of 19% at root initiation at 28 DAP compared to the control treatment (Fig. 5a). The IBA and M43 treatments positively affected the trigonelline content at 0 DAP and at root initiation at 28 DAP. The IBA showed an increase in trigonelline of 53% at 0 DAP and 32% at 28 DAP in relation to the control, while the M43 resulted in an increase of 61% at 0 DAP and 42% at 28 DAP compared to the control (Fig. 5b). Then, M21 treatment brought about a trigonelline peak at 7 DAP in the induction phase.

The IBA, M21, and M43 treatments showed higher content of total phenols than the control did in the induction rooting phase (Fig. 5c). The values corresponded to 6.7, 5.77, 6.32, and 4.81 mg g^−1^ of MF in the IBA, M21, M43, and control treatments, respectively. PPO activity was affected by IBA and M43 in the root extension phase (Fig. 5d). Application of IBA resulted in higher values at 47 and 69 DAP, and application of M43 resulting in higher values at 47 DAP compared to the control treatment.

### Principal component analysis

**Figure 6:**
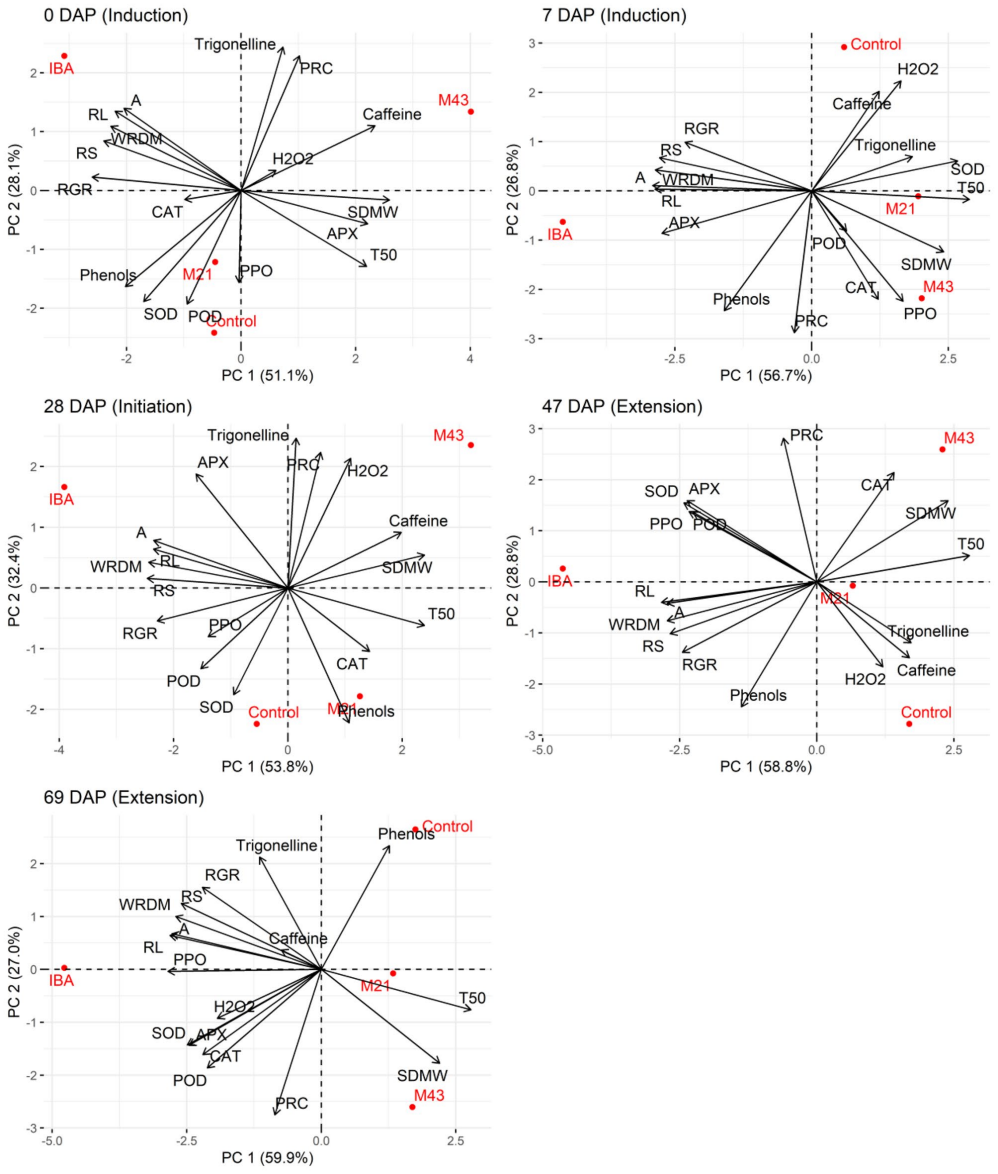
Principal component analysis in induction (0 DAP and 7 DAP), initiation (28 DAP) and extension (47 and 69 DAP) phasesas a function of IBA and melatonin application (M21 and M43) in *Coffea arabica* Catuai IAC-144 cuttings. The size and the direction of the vectors (arrows) represent the correlation between the variables and PC. *PEE* percentage of rooted cuttings, *RL* root length, *T50* time required to reach 50% of the maximum potential, *WRDM* root dry matter weight, *A* cumulative root area, *SDMW* shoot dry matter weight, *RS* root/shoot dry matter ratio, *RGR* relative growth rate, *H*_2_*O*_2_ hydrogen peroxide, *SOD* superoxide dismutase, *CAT* catalase, *APX* ascorbate peroxidase, *POD* peroxidase, *PPO* polyphenoloxidase, trigonelline and caffeine.

In Fig. 6, principal component analyses (PCA) are shown at 0, 7, 28, 47, and 69 DAP. The variables PRC, RL, A, T50, RGR, CAT, SOD, APX, POD, PPO, total phenols, caffeine, trigonelline, H_2_O_2_, SDMW, WRDM, and RS were used under application of melatonin M21, melatonin M43, IBA, and control treatments at 0, 7, 28, 47, and 69 DAP.

The PCA at 0 DAP showed that PC1 explained 48.8% of cumulative variance and PC2 28.6%, adding up to 77.4%. A separation of treatments by PC1 appears: where the IBA treatment is more related to the variables RL, RPA, A, WRDM, and RGR. M43 is more related to trigonelline, PRC, caffeine, and SDMW. Trigonelline showed a positive correlation with PRC in the M43 treatment.

A separation of treatments by PC1 appeared in the induction phase at 7 DAP. The APX, RL, A, WRDM, RPA, and RGR variables were associated with the IBA treatment; while the M43 treatment resulted in higher PPO, CAT and WRDM scores. Arrows in the same direction indicated positive correlations among PPO, CAT and SDMW, as well as for total phenols and PRC, which were associated with the M43 and IBA treatments (Fig. 6).

In the initiation phase at 28 DAP, there are positive correlations among PRC, H_2_O_2_, and trigoneline, as well as for caffeine and SDMW when M43 was applied. PC2 clearly showed that trigonelline, PRC, and H_2_O_2_ were associated with the IBA and M43 treatments (Fig. 6).

In the extension phase at 47 DAP, PC1 showed two groups of variables associated with the IBA treatment: RGR, RPA, WRDM, RL, and A, in contrast with PPO, POD, SOD, and APX. CAT and SDMW were grouped under the M43 treatment (Fig. 6).

At 69 DAP, most variables were associated with the IBA treatment by PC1: trigonelline, RGR, RPA, WRDM, A, RL, PPO, H_2_O_2_, SOD, APX, POD, and CAT. WRDM was associated with M43.

### Effect of melatonin and IBA on the dynamics of endogenous substances and enzymatic activity in the rooting and morphology phases

**Figure 7:**
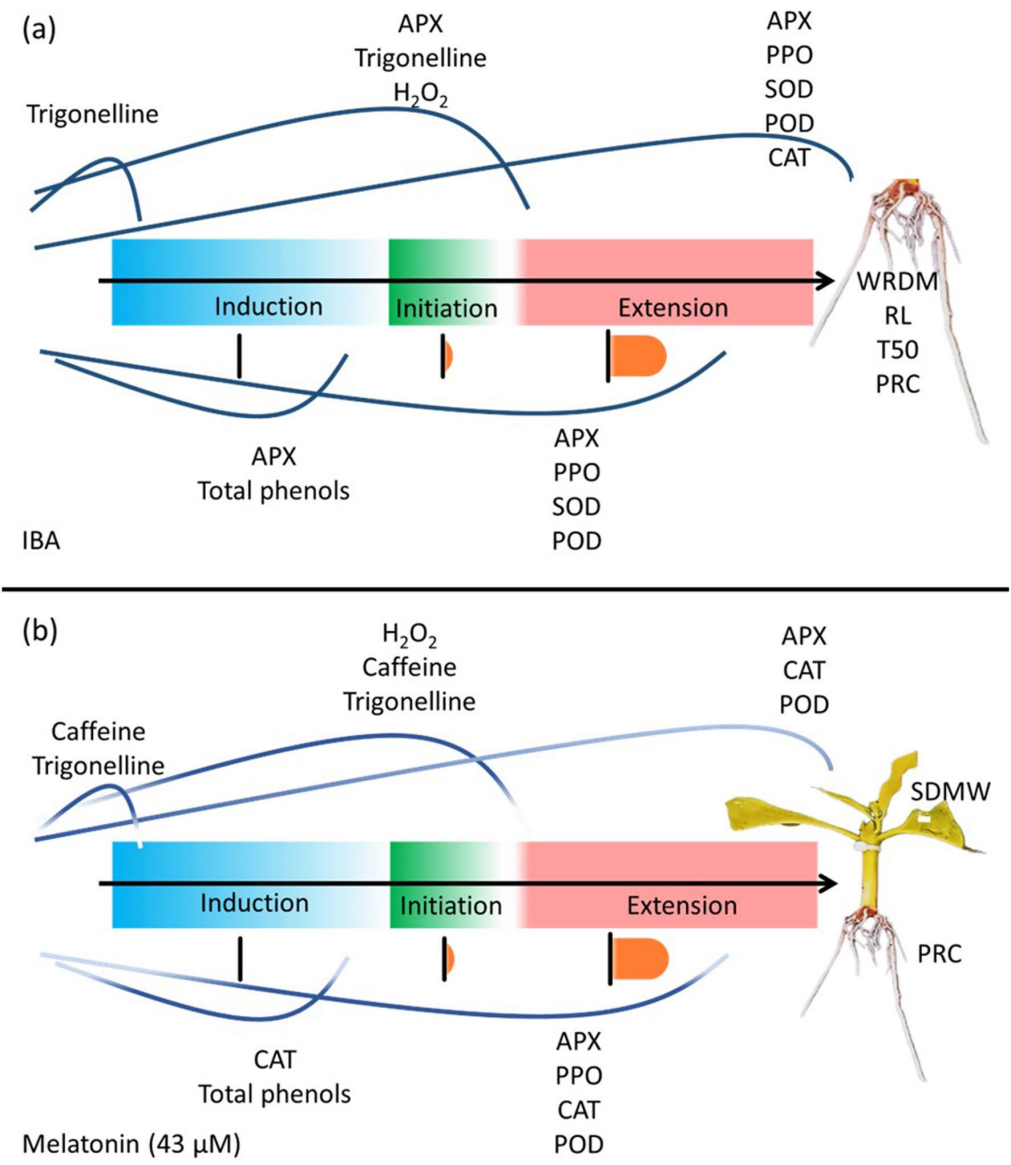
Dynamics of endogenous substances, enzymatic activity in the rooting phases and morphology in the treatments of IBA (**a**) and melatonin M43 (**b**). *PRC* percentage of rooted cuttings, *RL* root length, *T50* time required to reach 50% of the maximum potential, *WRDM* root dry matter weight, *SDMW* shoot dry matter weight, *H*_2_*O*_2_ hydrogen peroxide, *SOD* superoxide dismutase, *CAT* catalase, *APX* ascorbate peroxidase, *POD* peroxidase, *PPO* polyphenoloxidase, trigonelline and caffeine.

To analyze the dynamics of endogenous substances in the rooting stages, five points in time were evaluated: time zero at 0 DAP, induction at 7 DAP, initiation at 28 DAP, and extension at 47 and 69 DAP (Fig. 7). Cuttings of the Catuaí 144 cultivar under IBA, higher WRDM, RL, and PRC and lower T50 (Fig. 7a). The  melatonin M43 treatment led to higher PRC and SDMW (Fig. 7b).

Application of IBA resulted in peaks of total phenols in induction and H_2_O_2_ in initiation, in addition to trigonelline at time zero and initiation. APX activity was observed in root induction, initiation and extension. PPO, SOD, and POD increased in extension at 47 and 69 DAP, while CAT showed greater activity at 69 DAP (Fig. 7a).

Treatment with melatonin M43 gave rise to caffeine and trigonelline peaks at time zero and at initiation at 28 DAP. Total phenols peaked in the induction phase, while H_2_O_2_ peaked in initiation. CAT activity was reported in the initiation and extension phases. APX, PPO, and POD showed higher activities in the root extension phase (Fig. 7b).

## Discussion

In this study, it was possible to confirm effective methods for increasing rooting in C. arabica cuttings. As shown here, the application of M43 and IBA led to PRC above 90%. Thus, we consider that rooting was effective, as according to Vallejos-Torres et al.^20^, conversion greater than 85% allows large-scale production of *C. arabica* plantlets. Adventitious roots formation is a process involving morphological, physiological and biochemical changes, including the following phases: induction, initiation and extension^18^. This study showed that in the different rooting stages, trigonelline and caffeine concentrations were important inductors for discrimination of treatments that received the application of IBA and M43. This indicates that these alkaloids may be involved in the metabolism that resulted in higher percentage of rooting in these treatments. The highest concentrations of trigonelline and caffeine quantified at the beginning of the treatments (0 DAP) were positively correlated with higher final PRC. Since this quantification was carried out in samples collected shortly after the application of IBA and melatonin regulators, it appears that these regulators may induce relatively rapid effects on the secondary metabolism associated with the development of adventitious roots. According to Ashihara et al.^9^, alkaloids, such as caffeine and trigonelline, promote chemical defense against pathogens and herbivory. Furthermore, these alkaloids are also considered antioxidant molecules^8^. Trigonelline and caffeine can increase stress resistance and also act against pathogens in the area at the base of the cuttings, contributing to their survival. It should be noted that, in this study, the trigonelline peak was more consistent when IBA and M43 were applied, and the caffeine peak when M43 was applied. Indeed, the biosynthesis of alkaloids was stimulated by melatonin and auxin in *Leucojum aestivum* cultivated in vitro and the quality of the metabolic profile of alkaloids may vary according to the type of stimulant^23,24^.

An increase in trigonelline and caffeine concentrations was also observed in the control and M21 treatments, but only at 7 days of the induction phase. There was a delay in alkaloid peaks, which were not correlated with higher percentages of rooting and root morphological characteristics. This indicates that peak trigonelline and caffeine levels are involved in the induction phase of adventitious root development in control plants. However, the application of IBA and M43 may have accelerated these peaks and positively affected the initial induction phase. According to Wink and Witte^25^, alkaloids can be metabolized and the nitrogen of their molecules can be recycled. Therefore, we suggest that, in this study, trigonelline and caffeine may have been used after application; and in the early-induction phase they were mobilized as a nitrogen-source, which can explain the lower values of trigonelline and caffeine at 7 days in the IBA and M43 treatments.

In the initiation phase, at 28 DAP, the higher concentrations of trigonelline and caffeine also contributed to discrimination of the IBA and M43 treatments in relation to the control and M21, which were positively correlated with higher percentage of rooting and other morphological variables of the root and SDMW. Acidri et al.^4^ observed that trigonelline is found in all coffee plant organs, especially in those with an active meristem. In that phase, when the root primordia and meristems begin, there is intense cell division and, therefore, trigonelline and caffeine can affect the cell cycle. These alkaloids are secondary metabolites whose precursors are purine and pyridine nucleotides. Purines are key components of the cell energy system (e.g., ATP, NAD) and of signaling (e.g., GTP, cAMP, cGMP); and along with pyrimidines, they participate in the production of RNA and DNA^26^. In mungbean (*Phaseolus aureus*), trigonelline was found in high concentration in the embryonic axis during germination, as a result of higher biosynthetic activity and its transport from the cotyledons to the embryonic axis^16^.

In contrast, in the extension phase, when the rooting process had already completely occurred it appears that the application of IBA did not affect trigonelline content. However, higher concentrations of trigonelline and caffeine contributed to the formation of the component in the discrimination of the control and M43 treatments in relation to IBA. At the end of the extension phase, only trigonelline appears to be involved, as caffeine was in low concentrations.

This study also highlights the positive correlation between trigonelline and caffeine with H_2_O_2_ in all stages of adventitious root development. Corroborating these results, Chen et al.^27^ found that melatonin promotes lateral root formation triggered by the increase in H_2_O_2_. In addition, melatonin acted in signaling inducing the expression of cell cycle regulatory genes responsible for the formation of adventitious roots in *Medicago sativa*. Genetic evidence showed that two genes (AtCDKB1 and AtCDKB2) responsible for the formation of lateral roots and involved in the cell cycle were positively regulated by H_2_O_2_. However, when an NADPH oxidase inhibitor (Diphenyleneiodonium—DPI) was used and/ or H_2_O_2_ scavenger (Dimethylthiourea—DMTU) both gene expression and lateral root formation were impaired^27^.

Li et al.^28^ also demonstrated that H_2_O_2_ acts as a promoter during adventitious root formationin *Vigna radiata* cuttings. H_2_O_2_ acts by regulating the expression of genes involved in many cellular processes associated with stress response, cellular redox homeostasis and response to oxidative stress, secondary metabolism, cell wall loosening and modification, nutrient and energetic processes, cellular component movement, transcription, DNA synthesis, and cell cycle and transcription factors (TFs), as well as plant hormone signaling pathways.

In general, the correlations between trigonelline and caffeine with antioxidant enzymes were observed according to the root development stage in this study. High alkaloid concentrations were observed soon after IBA and M43 application, which was not found for enzymes. Consequently, a negative correlation was observed at 0 DAP between trigonelline and caffeine with the enzymes SOD, POD, PPO, and CAT. Thus, IBA and M43 seem to bring about a faster response to alkaloids than antioxidant enzymes. This negative correlation discriminating IBA and M43 from the other treatments is also observed at initiation at 28 DAP and at the beginning of extension at 47 DAP. It is possible that with the antioxidant activity of these alkaloids there is less activity of antioxidant enzymes in these phases. Alkaloids have antioxidant activity and free radical scavenging, acting as hydrogen and electron donors^1^.

However, the joint action of enzymatic and non-enzymatic antioxidants at the end of the extension phase (68 DAP) was positively correlated with higher PRC and greater root development in cuttings treated with IBA. We found that the increase in the activity of antioxidant enzymes, mainly from initiation induced by IBA and M43, was also associated with better root development. Therefore, we assume that the antioxidant system can modulate the adventitious root formation process by controlling the cellular redox state. This is corroborated by Vilasboa et al.^29^, in which the greater rooting capacity of the *Eucalyptus grandis* species was associated with greater antioxidant enzyme activity and greater flavonoid content. These characteristics may affect ROS-based signaling and phytohormone homeostasis in cuttings, thus impacting adventitious root development.

This paper also showed that the phenolic compounds in the induction phase assisted root formation under the application of melatonin M43 and IBA. The connection of phenolic compounds with rooting has already been reported in previous research, in which di and polyphenols acted in antioxidant defense and protection of auxins against decarboxylation^2^. In *C. arabica* plants, the action of phenolic compounds on rooting has not yet been well established.

The results of this study suggest that melatonin was correlated with higher shoot dry matter weight, indicating that it stimulates greater allocation of dry matter to shoots than to roots, which is also shown by the lower RS. According to Behling et al.^30^, the relationship between leaf dry matter and root dry matter reflects an approximate source and sink relationship. At 69 DAP, the M43 treatment provided a larger source area, which may be reflected in a higher CO_2_ content being fixed and redistributed throughout the plant. In addition, as the seedling is not yet ready to go to the field at 69 DAP, more studies need to be carried out to see the consistency of the RS ratio up to the time the plantlets will be ready for transplanting in the field.

With regard to enzymatic activity in the induction phase, CAT correlated positively with PRC, suggesting that in *C. arabica* cuttings its activity may be important for adventitious root formation under application of melatonin M43. APX, however, was stimulated by the IBA treatment from the induction phase to the extension phase. There are still few studies reporting the relationship between changes in CAT and APX activity and their functions during adventitious root formation^31^. Racchi et al.^32^ found strong activity of the CAT-2 isoform in rooted oak microcuttings, suggesting that this isoform is a protein specifically related to rooting. In this study, CAT and APX activity correlated negatively with H_2_O_2_ content in the induction phase, suggesting that both enzymes play an important detoxification role in that phase. Finally , the correlations between trigoneline and caffeine with phenols and antioxidant enzymes reveal different profiles, depending on the phases, showing the complexity of processes that may be involved in the redox balance during adventitious root development in *C. arabica* cuttings.

In conclusion, IBA and melatonin increase trigonelline and caffeine during the induction and initiation of adventitious roots in *Coffea arabica* cuttings, which is correlated with a higher percentage of rooted cuttings. Since the *C. arabica* cultivars have variations in caffeine and trigonelline concentrations, these alkaloids could explain, at least in part, the variations in rooting capacity between coffee cultivars and the inconsistent success of the available techniques. Therefore, this study opens the possibility of exploring the correlation of these parameters with rooting capacity. This may contribute to the establishment of models for early prediction of rooting recalcitrance, thus improving clonal selection and propagation in breeding programs.

## Materials and methods

### Experiment site

The experiment was conducted from January to March 2020 in the Plant Physiology sector of the Biology Department of the Federal University of Lavras (Universidade Federal de Lavras - UFLA), Lavras, MG, Brazil (21º13′40’’S and 44º57′50’’ W, at 960 m asl). Climate classification is type Cwa (Köppen and Geiger). The greenhouse was irrigated every 5 min for 30 s from 6:00 a.m. to 9:00 p.m. in order to maintain relative humidity above 85%. The temperature ranged from 18 to 28 °C.

### Plant material

Seedlings of the Catuaí IAC-144 cultivar approximately one year and eight months of age were used. The seedlings were provided by the Coffee Growing Sector of UFLA. The use of coffee tree parts complies with international, Brazilian and institutional guidelines.

### Experimental procedures

The shoot apex of the coffee plants was pruned and curved to force the sprouting of new orthotropic branches. The cuttings were approximately 5 cm long, with a bevel cut at the base and the cut in the middle of the leaves. Each cutting had only one pair of leaves. The cuttings were treated with 0.05% sodium hypochlorite for 10 min and washed with water^33^. The cuttings were then separated and immersed in solutions with 21 µM melatonin (M21), 43 µM melatonin (M43), or 7380 µM indole-3-butyric acid (IBA) for 3 min in the absence of light. The concentrations used had been defined from preliminary testing. Cuttings without any treatment with inducers were used as controls. After the treatments, the cuttings were inserted in tubes with substrate. Two types of tubes were used: transparent with a volume of 70 cm^3^ and black with a volume of 120 cm^3^. The transparent tubes were placed inside the black tubes with a Styrofoam ring, and both tubes were attached to a tray with an inclination of 13.5°. This system was created to aid in cumulative rooting and root growth periodically. The BASAPLANT® (Artur Nogueira, Brazil) commercial substrate for vegetables and sand were used at a proportion of 6/4, respectively.

### Experimental design

A completely randomized design was used with four replications and four treatments [control, 21 µM melatonin (M21), 43 µM melatonin (M43) and 7380 µM indole-3-butyric acid (IBA)] and each plot was composed of 10 experimental units for the phytotechnical variables. For the biochemical variables, however, a split-plot arrangement was used, in which the plot levels were composed of the control, M21, M43 and IBA treatments, and the subplots composed of the evaluation times (days after planting 0, 7, 28, 47 and 69 DAP); five cuttings per experimental plots were used. The 0 DAP (0 days after planting the cuttings) designates the time immediately after treatment of the cuttings with the inducers, before they were planted in the tubes with the substrate. As found in our preliminary study, the root induction phase for *C. arabica* cuttings lasted 20 days. Therefore, we performed the sampling at 0 and 7 days of evaluation. Accordingly, the initiation phase - the beginning of the root primordium - was from 21 to 34 DAP. Consequently, sampling was performed at 28 DAP. The extension phase - extension of the root primordium - was from 35 to 69 DAP. Sampling in this phase was performed at 47 and 69 DAP.

### Phytotechnical variables

To calculate the percentage of rooted cuttings (PRC), the number of rooted cuttings (NRC) and the number of total cuttings (NTC) were used according to the equation NRC/NTC*100. Root length (RL) was measured using a graduated ruler (cm). Relative growth rate (RGR) was quantified according to Floss^34^ usingthe following equation:

$$\begin{aligned} {\text{M1}} = {\text{Length}}\;{\text{of}}\;{\text{the}}\;{\text{first}}\;{\text{collection}}; \\ & {\text{M2}} = {\text{Length}}\;{\text{of}}\;{\text{the}}\;{\text{last}}\;{\text{collection}}; \\ & {\text{T1}} = {\text{DAP}}\,{\text{of}}\;{\text{the}}\;{\text{first}}\;{\text{collection}}; \\ & {\text{T2}} = {\text{DAP}}\;{\text{of}}\;{\text{the}}\;{\text{second}}\;{\text{collection}}; \\ & {\text{nl}} = {\text{natural}}\;{\text{logarithm}}. \\ \end{aligned}$$

The root dry matter weight (WRDM) and shoot dry matter weight (SDMW) were determined from the fresh matter, that remained 72 h in a forced air circulation oven at 65°C. The dry matter was then weighed on a digital scale. The root/shoot dry matter ratio (RS) was obtained by dividing the WRDM by the SDMW.

To quantify the T50, a sigmoid equation (ŷ = α /(1 + e^ − ((x − x_0_) /b)) of three terms was used, as described by Romano et al.^35^. The x_0_ (T50) represents the point at which the curve reaches 50% of the maximum potential of rooted cuttings. Cumulative root area (*A)* is calculated from the same equation; the area under the sigmoid curve from 0 to 76 days is a definite integral of the function and represents the total percentage of rooted cuttings.

### Biochemical variables

The phloem was peeled from fresh stems from the base up to two cm; this material was cut into smaller segments, immediately frozen in liquid nitrogen and kept at − 80° C until analysis. The analyses at 0 DAP were performed immediately after the cuttings were treated with the inducers.

### Quantification of hydrogen peroxide (H_2_O_2_)

The method described by Velikova et al.^36^ was used. For extraction, 100 mg samples of fresh stems were macerated in liquid nitrogen with 50% polyvinylpolypyrrolidone (PVPP) and homogenized with 1500 μL of trichloroacetic acid (TCA—0.1%). The samples were centrifuged at 13,000 g for 10 min at 4 °C and 45 μL of the supernatant was used for reaction with 45 μL of potassium phosphate buffer (10 mM, pH 7.0) and 90 μL of potassium iodide (1 M) . The absorbance reading was performed at 390 nm in duplicate and the H_2_O_2_ levels were quantified by means of a standard curve with the stock solution of H_2_O_2_ at 250 μM.

### Quantification of total phenols

Samples of 100 mg of fresh stem were macerated in liquid nitrogen with 50% PVPP and homogenized with 500 μL of extraction buffer (methanol—99%; HCl—1%) in 2 mL microtubes. Subsequently, the extract was placed on ice, stirred for 4 h in the dark^37^, and centrifuged at 13,000 g for 30 min at 4ºC. The supernatant was collected and 250 μL of buffer solution was added to the decanted material, which was centrifuged again at 13,000 g for 30 min at 4ºC and the second supernatant was removed. The second supernatant (2 μL) was used for the reaction with 2 μL of pure folin, 9 μL of anhydrous sodium carbonate (220 mg mL^−1^) and 307 μL of deionized water. The reaction was incubated for 30 min in a water bath at 40°C. The reading was carried out in a spectrophotometer at 765 nm, and the levels of Gallic acid (GA) were quantified by means of a standard curve with AG stock solution at 1 mg mL^−1^.

### Extraction and quantification of enzymes

For extractions of enzymes [superoxide dismutase (SOD), catalase (CAT), ascorbate peroxidase (APX), peroxidase (POD), and polyphenoloxidase (PPO)], 100 mg samples of fresh stems were macerated in liquid nitrogen with 50% PVPP and homogenized with 1.5 mL of potassium phosphate buffer (400 mM, pH 7.8) containing 10 mM ethylenediaminetetraacetic acid (EDTA) and 200 mM ascorbic acid^38^. The extract was centrifuged at 13,000 g for 10 min at 4 °C and the supernatant was collected to quantify enzyme activity. The protein dosage was determined using the Bradford method^39^. All enzymes activity were performed in triplicates.

### SOD activity

SOD activity was quantified according to the methodology described by Giannopolitis and Ries^40^, which is based on the ability to inhibit the photoreduction of nitrotetrazolium blue (NBT). An aliquot of the sample was added to the incubation medium containing 100 μL of potassium phosphate buffer (100 mM, pH 7.8), 40 μL of L-methionine (70 mM), 3 μL of EDTA (10 mM), 15 μL of NBT (10 mM), 2 μL of riboflavin (0.2 mM) and 30 μL of deionized water.

The reaction was carried out under fluorescent light (20 W) for seven minutes, while the blank consisted of deionized water plus the incubation medium. The formation of blue formazan, derived from the reduction of NBT, was determined by reading the spectrophotometer at 560 nm. One SOD unit was defined as the amount of enzyme needed to inhibit NBT reduction by 50%.

### CAT activity

CAT activity was quantified according to Havir and Mchale^41^. Aliquots of the enzyme extract were added to the incubation medium, containing 90 μL of potassium phosphate buffer (200 mM, pH 7.0) and 72 μL of deionized water and incubated at 30 °C in a water bath. At the time of reading, 9 μL of H_2_O_2_ (250 mM) was added. Then, CAT activity was measured by the decrease in absorbance at 240 nm every 15 s over a period of 3 min, monitored by consumption of hydrogen peroxide.

### APX activity

APX activity was measured in a medium composed of the enzyme extract, 90 μL of potassium phosphate buffer (100 mM, pH 6.0), 9 μL of ascorbic acid (10 mM) and 63 μL of deionized water. This was incubated at 30°C in a water bath, 9 μL of H_2_O_2_ (2 mM) was added^42^. Ascorbate oxidation was read in the spectrophotometer at 290 nm, every 15 s for 30 min^43^ (Nakano; Asada, 1981). The molar extinction coefficient used was 2.8 mM^−1^ cm^−1^.

### POD activity

POD activity was determined according to the methodology of Neves^44^. Aliquots of the enzyme extract were added to the reaction medium composed of 180 μL of sodium phosphate buffer (100 mM, pH 6.0), 60 μL of guaiacol (0.8%), 60 μL of H_2_O_2_ (0.9%) and 50 μL of deionized water. The sodium phosphate buffer was incubated at 30 °C. The reading was performed in a spectrophotometer at 470 nm, every 15 s over a period of 2 min. The molar extinction coefficient used was 26.6 mM^−1^ cm^−1^.

### PPO activity

PPO activity was determined according to the methodology adapted from Kar and Mishra^45^. Aliquots of the enzyme extract were added to the reaction medium composed of 200 μL of sodium phosphate buffer (70 mM, pH 7.0) and 40 μL of catechol (100 mM). The reaction medium was incubated at 30°C for 5 min before adding the extract. The reading was performed in a spectrophotometer at 410 nm, every 13 s over a period of 1.3 min. The molar extinction coefficient used was 1.235 mM^−1^ cm^−1^.

### Bioactive compounds

Caffeine and trigonelline were quantified by high performance liquid chromatography (HPLC), according to the methodology of Malta and Chagas^46^. The extract was taken from 300 mg of fresh stem macerated in nitrogen with 50% PVPP, which was placed in 10 mL of distilled water and then incubated for 3 min. The extract was filtered through common filter paper with a 0.45 μm membrane. Quantification was performed on a chromatograph (Schimadzu®, Kyoto, Japan) with a diode array detection system (model SPD-M43A), a Discovery C18 chromatographic column (250 × 4.6 mm, 5 μm) and a wavelength of 272 nm. The mobile phase consisted of methanol: water: acetic acid (20:80:1), with a flow rate of 1 mL min.^−1^.

### Statistical analysis

Statistical analyses of the data were performed on the R version 4.0.5 platform. Normality and homogeneity tests, Shapiro-Wilk and Bartlett, respectively, the test F (*p* < 0.05), and the Duncan test at 5% probability were conducted to compare means, using the ExpDes.pt statistical package^47^. Principal component analysis (PCA) was also performed with the prcomp function of the R software and the factoextra package^48^. The SigmaPlot®, version 12.3 software was used to fit the sigmoid and exponential model.

## Data Availability

Datasets generated during and/or analyzed during this paper are not publicly available for data security reasons, but are made available by the corresponding author upon reasonable request.
